# Career experts’ conceptions of innovation in career development

**DOI:** 10.1007/s10775-021-09509-9

**Published:** 2021-10-08

**Authors:** Jaana Kettunen

**Affiliations:** Finnish Institute for Educational Research, University of Jyväskylä, P.O. Box 35, 40014 Jyvaskyla, Finland

**Keywords:** Career development, Innovation, Phenomenography

## Abstract

This article reports the findings from a phenomenographic investigation into career experts’ conceptions of innovation in career development. The results show that conceptions of innovation in career development varied from (1) initiating service, (2) developing demographic-based programmes, (3) professionalising the sector to (4) exploiting cross-sectoral synergies. The findings give us a more profound understanding of critical aspects that may have an important role in improving innovation in career development.

## Résumé

**Conceptions d’experts de l’orientation des innovation dans le domaine du développement de carrière** Cet article rapporte les résultats d'une étude phénoménographique sur les conceptions des expert·e·s de carrière en matière d'innovation dans le développement de carrière. Les résultats montrent que les conceptions de l'innovation dans le développement de carrière varient de (1) l'initiation du service, (2) le développement de programmes pour des populations spécifiques, (3) la professionnalisation de secteurs à (4) l'exploitation des synergies intersectorielles. Les résultats nous donnent une compréhension plus profonde des aspects critiques qui peuvent avoir un rôle important dans l'amélioration de l'innovation dans le domaine du développement de carrière.

## Abstract (de)

**Die Vorstellungen von Karriereexperten über Innovation in der Karriereentwicklung** Dieser Artikel berichtet über die Ergebnisse einer phänomenographischen Untersuchung zu den Vorstellungen von Laufbahn-Experten über Innovation in Programmen der Laufbahnentwicklung. Die Ergebnisse zeigen, dass die Vorstellungen von Innovation in der Laufbahnentwicklung von (1) der Initiierung von Dienstleistungen, (2) der Entwicklung von Programmen auf Basis demografischer Informationen, (3) der Professionalisierung des Sektors bis hin zu (4) der Nutzung von sektorübergreifenden Synergien variieren. Die Ergebnisse geben uns ein tieferes Verständnis für kritische Aspekte, die eine wichtige Rolle bei der Verbesserung der Innovation in der Laufbahnentwicklung spielen können.

## Resumen

**Concepciones de los expertos sobre la innovación en el desarrollo de la carrera** Este artículo presenta los resultados de una investigación fenomenográfica sobre las concepciones de los expertos en carreras profesionales sobre la innovación en el desarrollo de la carrera. Los resultados muestran que las concepciones de la innovación en el desarrollo de la carrera varían desde (1) la iniciación del servicio, (2) el desarrollo de programas de base demográfica, (3) la profesionalización del sector hasta (4) el aprovechamiento de las sinergias intersectoriales. Los resultados nos permiten comprender más profundamente los aspectos críticos que pueden tener un papel importante en la mejora de la innovación en el desarrollo de la carrera.

## Introduction

The world of work has changed considerably in recent decades as a consequence of multiple factors that include globalisation, digitalisation and automation (e.g. Cedefop, 2016; Organization for Economic Co-Operation and Development [OECD], 2019; Patton & McMahon, 2015). Traditional occupations and work methods are changing fast (e.g. Brynjolfsson & McAfee, 2014; Frey & Osborne, 2017), and individuals are increasingly required to cope with serial work and life transitions, including periods of under- or over-employment, multiple career changes and returns to education (e.g. Amundson, 2005; Hirschi, 2018; McMahon & Tatham, 2008; OECD, 2019) as previously non-standard forms of work—temporary, short-term and agency-based work and self-employment—become more widespread (e.g. Dølvik & Steen, 2018; International Labour Organization [ILO], 2016; Eurofound, 2017). In light of these changes, the individuals must embrace continuous learning and flexibility to maintain their employability and create their own opportunities (e.g. Amundson, 2005; Savickas et al., 2009).

Career development services are perceived to be one accepted way of assisting individuals to make well-informed and realistic decisions about education, training and occupational choices and acquire lifelong career management skills (Cedefop, 2011; European Council, 2004, 2008; OECD, 2004). In this context of rapid change, the career development sector must innovate and evolve to meet the challenges of this dynamic connected economy (e.g. Barnes et al., 2020; Lee & Johnston, 2001; OECD, 2004). Calls to enhance professionalism within the career development sector are increasing (e.g. Cedefop, 2009; OECD, 2010; Sienkiewicz, 2012). However, the OECD (2004) and Cedefop (2009) have found little evidence of systematic and continuous professional development for career professionals. Over the past decades, public services have been among the main adopters of information and communication technologies (e.g. Van Reenen et al., 2010), and technology is having an impact on traditional career guidance services. As interactive technology continues to grow in popularity and influence, it is increasingly necessary to align new technologies more closely with career services and associated professional practices (e.g. Bimrose et al., 2010; Kettunen, 2017; Sampson et al., 2020).

There is increasing recognition that innovation is one of the a key means by which public services can become more effective, more efficient or more legitimate (Windrum & Koch, 2008). As countries differ widely in the development and delivery of career services, the idea of innovation is necessarily relative, where career development services are scarce or non-existent, for example, almost any initiative can be regarded as innovative (Zelloth, 2009). In general, innovation can be defined as ‘[…] the successful introduction of something new and useful, for example, introducing new methods, techniques, or practices or new or altered products and services’ (Dasgupta & Gupta, 2009, p. 205). According to Van de Ven (1986, p. 2), however, ‘as long as the idea is perceived as new to the people involved, it is an “innovation”, even though it may appear to others to be an imitation or something that exists elsewhere’. In any event, innovation is about problem-solving and addressing challenges in life and society that are complex, multi-faceted and require a creative approach (Hautamäki & Oksanen, 2016). Although it seems that for many public services, change and innovation are mainly top-down, planned and formalised processes (e.g. European Commission, 2011; Hartley, 2005), recent literature points to the rise of collaborative innovation in the public sector (e.g. Bommert, 2010; Torfing, 2019). This multi-actor collaboration is envisaged as taking place inside public administrations, between members of different public bodies, but also with private partners. Many scholars have highlighted that complex problem-solving benefits from diverse perspectives (Van der Vegt & Janssen, 2003), multi-professional collaboration (Kettunen & Felt, 2020) and engagement with multiple stakeholders (e.g. Barns et al., 2020; Pittaway et al., 2004), integrating diverse knowledge bases, behaviours and ways of thinking.

In career development contexts, innovation may involve new theories, methods, services, practices, techniques, tools, or institutional structures to produce a significant change in career service delivery. For example, technological innovations like the expansion of Internet, mobile phone and social media use present new opportunities for people to give and receive career support (e.g. Flederman & Watts, 2014; Hooley et al., 2010; Kettunen et al., 2015; Sampson et al., 2020). In an analysis of career theory and practice worldwide, Amundson (2005) identified theoretical innovations grounded in constructivism, systems theory, action theory and fragmentary developments highlighting the importance of paradoxical thinking. Innovations in career development practice included a greater emphasis on active engagement, more holistic methods and an increased emphasis on counselling efficacy (Amundson, 2005). Drobnic (2019) noted that recent innovations in career guidance on labour market developments have driven an increased emphasis on the career guidance client's autonomy, which necessarily positions the counsellor in a non-authoritative role. According to Barns et al. (2020), new and innovative guidance practices and tools aim to provide seamless service delivery across the individual’s life course. This more integrated lifelong approach emphasises user centrality, individually tailored service provision and enhanced networking based on digital technologies (Barns et al., 2020).

Innovation studies in the area of career development are rare and there is an apparent research gap that this study aims to narrow. The present study explores innovation in the career development sector as perceived by international career development experts. The aim was to identify and describe variation of qualitatively different conceptions of the phenomenon. The study was guided by the following research questions: (1) What are career development experts’ conceptions of innovation in career development? and (2) What are the critical aspects that differentiate the qualitatively varying ways of understanding career development innovation? The ultimate aim of describing the variation in conceptions is to expand understanding of the critical aspects that may play important role in improving innovation in career development.

## Methods

The study employed a phenomenographic approach, which investigates individual variations in experiencing or understanding a given phenomenon (Bowden & Walsh, 2000; Marton, 1981; Marton & Booth, 1997). Phenomenography assumes that a particular phenomenon can be experienced in only a limited number of ways, and that these are logically related. The primary outcome of any phenomenographic analysis is a structured set of logically related categories describing qualitative variations in ways of experiencing or understanding the phenomenon in question at a collective level (Marton, 1986). According to Marton and Booth (1997), these categories of description should meet the following three quality criteria: (1) each category should describe a distinct way of conceiving the phenomenon, (2) the logical relationship between categories should be hierarchically represented, and (3) variation across the sample should be described by a limited number of parsimonious categories. Steps taken to meet these criteria in the present study are described in the data analysis section.

Previous phenomenographic studies of career development have explored issues that include career practitioners’ conceptions of social media (Kettunen et al., 2013); ethical practice in social networking (Kettunen & Makela, 2019); leadership and management in guidance and counselling networks (Nykänen, 2011); the role of ICT in relation to national guidance policies (Kettunen et al., 2016); and challenges in implementing ICT in career services (Kettunen & Sampson, 2019).

### Participants and the study context

The participants in this study were designated governmental and non-governmental representatives from 33 countries attending the International Symposium for Career Development and Public Policy (hereafter ICCDPP Symposium). This ninth Symposium invited delegates to share their experiences in the development of national strategies for career development and to explore opportunities for ongoing networking (Watts et al., 2014) to harness the benefits of sharing international practice and policy development (Kettunen & Sampson, 2019; Watts, 2005). Respondents represented policymakers, policy developers and influencers, national authorities, international institutions, managers of national career guidance services, leaders of national and international associations of guidance practitioners, employers representatives, private enterprises and researchers and trainers of career practitioners, thus covering the entire career development community. National teams that answered the questionnaire consisted of 134 delegates, of whom 71 were women and 63 were men; 37 were government employees in ministries of education and employment; 40 were civil servants working in the education and labour sectors; 52 represented national guidance forums, centres or associations; and 5 worked in the private sector. The data collection was a part of the preparation for the symposium in Tromssa, Norway, in June 2019, which addressed issues of access, integration and innovation in an uncertain future.

### Data collection

The survey data were collected using open-ended questions that asked participants to provide written responses at a national level as country teams. Since the primary focus was on capturing the richness of the collective experiences of a relatively large number of participants located around the world, the open-ended survey method constituted an appropriate means of data collection for this phenomenographic research. Although the written responses provided little or no opportunity for follow-up questions, this approach has been successfully applied in a number of previous phenomenographic studies (e.g. Hathaway & Fletcher, 2018; Kettunen & Sampson, 2019; Loughland et al., 2002; Tynjälä, 1997).

The Symposium coordination unit emailed a link to the online survey in November 2018, and responses (one response per team) were received from the following 33 country teams: Austria, Cambodia, Canada, Chile, Croatia, Denmark, Egypt, England, Estonia, Finland, France, Germany, Ghana, Hungary, India, Ireland, Japan, Korea, Kosovo, Luxembourg, Mongolia, Norway, Qatar, Scotland, Serbia, Singapore, Slovenia, Sri Lanka, Switzerland, Syria, the Netherlands, Tunisia and the United States. Each country delegation of one to six members was asked to provide a country-specific response to the following four open-ended questions: (1) What are the key innovations or ways that career development programmes and services have changed over the last 10 years? (2) How do national policies and initiatives, where they exist, ensure and support the development of innovation in career development services provision? (3) What new, innovative and promising interventions in career development programmes and services is your country planning for the future? and (4) How is the training and continuing professional development (CPD) of careers professionals encouraging innovation and taking account of new evidence as well as changes in technology and the labour market? Responses ranged in length from half a page to five pages, averaging two pages. In total, the data yield 62 pages of text (A4, single spaced).

### Data analysis

Data analysis was conducted using a phenomenographic approach (Åkerlind, 2005b; Kettunen & Tynjälä, 2017; Marton & Booth, 1997; Marton & Pong, 2005), following the guidelines and examples provided by Åkerlind (2005a, 2005b), Bowden (2000a), and Bowden and Green (2005). The analysis involved three phases. The first phase sought to identify and describe conceptions of career development innovation in general terms, ‘bracketing’ presuppositions and personal understandings to maintain an open mind (Ashworth & Lucas, 2000; Marton & Booth, 1997). The written responses were considered as a whole and re-read a number of times to identify underlying foci and intentions expressed in them. To improve accuracy in the interpretation, the transcript was read as a whole or in large sections (Åkerlind et al., 2005). To identify key relationships and distinct characteristics, subsequent readings focussed on similarities and differences in the meanings expressed. By comparing and contrasting these similarities and differences, a draft set of descriptive categories of collective meaning was gradually developed, defined and named.

The second phase of the analysis sought to delineate the logical relationships among the various categories. Themes that ran through and across the data were identified and used to structure the relationships within and between categories (Åkerlind, 2005a). The aim was to distinguish between more and less complex ways of seeing the phenomenon (Åkerlind, 2005a; Marton & Booth, 1997), to highlight its different aspects. Throughout this phase, the initial categories of description were further elaborated, fixed and defined according to the characteristic features of each category, with constant reference to the data.

To ensure robustness, the data were initially analysed by the author, who then sought a second opinion from a colleague. The categories and their structures were discussed and revised during several such meetings, so ensuring that the interpretations were valid. Research colleagues acted as critical friends, probing the category candidates and their critical aspects and seeking justifications from within the transcripts. As Bowden (2000b) emphasised, this group process makes it less likely that analysis will cease prematurely, and helps to ensure loyalty to the data minimising the researcher’s individual perspective and potential for bias. Iterative re-reading and re-drafting continued until saturation—that is, until the process failed to produce any further significant changes in the categories of description (Bowden & Green, 2010).

The final phase of analysis sought to ensure that the categories of description met the quality criteria mentioned earlier, as specified by Marton and Booth (1997): (a) that each category described a distinct way of experiencing the phenomenon; (b) that the logical relationships between categories were hierarchical; and (c) that in describing variation across the sample, the categories were parsimonious and limited in number.

## Results

Analysis of the data revealed four distinct categories of description that reflected career development experts’ conceptions of innovation in career development (Table 1). Innovation in career development was conceived as: (1) initiating service, (2) developing demographic-based programmes, (3) professionalising the sector, and (4) exploiting cross-sectoral synergies. The differences between the categories appeared along six dimensions of variation that included: innovative initiatives, development emphasis, rationale for developing ICT use, delivery of professional development and realisation of career education.

**Table 1:** Career experts’ conceptions of innovation in career development

Dimensions of variation	Categories			
	Initiating services	Developing demographic-based programmes	Professionalising the sector	Exploiting cross-sectoral synergies
Innovative initiatives	Desired	Initiated	Stimulated	Supported
Development emphasis	Services	Programs	Professionalism	Quality
Rationale for developing ICT use	Raising awareness/ promoting	Widening access	Effectiveness	Efficiency
Continuing professional development	Acknowledged need	Ad hoc training	Short courses	Sustained programme
Delivery of professional development	Not yet planned	Face-to-face delivery	Blended delivery	Online delivery
Realisation of career education	Piloted	Introduced	Integrated	Timetabled

Each category is described in more detailed below, along with excerpts from relevant written responses to illustrate key aspects of the categories. It is important to note that this categorization represents collective rather than individual or country-specific conceptions of innovation in career development.

### Description of the categories

#### Category 1: Initiating services

In this first category, innovation is conceived as initiating career development services. Innovative initiatives were seen as *desired* with a general ambition to innovate. Focus on development was placed on setting up *services* that have been lacking as a result of political instability or the short history of career services in that country. A key rationale for developing the use of ICT was to *promote* the value of career development and related services by providing more information to the public about what professional careers support can offer them and what to look for when seeking access.

Projects have a fundamental ambition to innovate and develop something new.

[National body] is piloting career guidance and counselling services for selected schools.

[Country] is planning to develop online platform to promote career guidance and counselling.

In this category, country teams indicated that there was an *acknowledged need* and/or initial plans for creating continuing training specific to career services. Decisions about how such training would be delivered were *not yet planned*. Here countries had started *piloting* career education curriculum in selected schools or educational institutions.

The state will set up a continuing training program for career guidance counsellors.

The technical education in [country] started to pilot a career development curriculum.

#### Category 2: Developing demographic-based programmes

In this second category, innovation was conceived as developing demographic-based career development programs. Innovation was mainly *initiated* from the top-down by public organisations (i.e. based more or less on political decision-making). Focus on development was placed on designing career-related *programmes* that meet the specific needs or requirements of a particular group of people. A key rationale for developing the use of ICT was to *widen* and extend *access* to career-related information, principally through digital platforms and mobile application.

Currently the innovation in career development services is very much related, and/or the result of national policies and initiatives.

Investments in demographic-based programs responding to the needs of an ageing population, foreign trained professionals, refugee newcomers and indigenous youth have driven new career development approaches, methodologies and service delivery models.

Access to information has been greatly facilitated with the creation of platforms and digital resources . . .

In this category, continuing professional development training is offered on an ad hoc basis but remained confined to a limited number of courses and programmes which take place in *face-to-face* settings. Career guidance program and/or activities were *introduced* as part of the to school *curriculum*, but participation in these programmes and/or activities was voluntary rather than mandatory.

There were ad hoc trainings for a small number of teachers in schools and for those employed as career centre staff.

School-based career guidance program and activities are introduced to school curricula.

#### Category 3: Professionalising the sector

In this third category, innovation in career development was conceived as professionalising the sector. Innovation initiatives were *stimulated* by top-down demands empowering bottom-up innovation among key regional actors. Development focuses on career practitioners’ *professionalism* in terms of their training and qualifications. Country teams indicated that professional competencies and competency standards were under revision or would be revised. A key rationale for developing the use of ICT was to improve the *effectiveness* of the services provided by extending opportunities to provide distance career services, using synchronous real-time phone- or chat-based communication.

The counties were given quite a lot freedom . . . and local innovation was stimulated.

Professionalisation of all those involved in career development is critical. The introduction of a new higher apprenticeship scheme for career development will offer a new professional pathway, making training more accessible for new practitioners. This is specifically important as the sector is currently experiencing a skills shortage.

Career development professional competencies and competency standards will be expanded and revised.

The main driver regarding the governance has been the aim to make effective use of resources . . . [This includes] the use of several digital solutions to support citizens career development (chat service online; the use of digital learning environments).

Here continuing professional development and *short* or refresher *courses* on new information or new methods were conducted on a regular basis. Beyond the traditional face-to-face model, these courses were delivered in *blended* mode combining face-to-face learning activities and online learning components. Career development education was now *integrated* in the curriculum and delivered through series of themes taught *across different subjects*.

Constant refresher training has been conducted.

The blended CPD approach . . . offers an opportunity for practitioners to enhance their digital skills.

Career guidance became an integral part of the [country name] curriculum framework, involving its offer in the curriculum.

#### Category 4: Exploiting cross-sectoral synergies

In this forth category, innovation in career development was conceived as exploiting cross-sectoral synergies. Innovative initiatives were *supported* to achieve common strategic development goals at country level by enhancing cross-sectoral collaboration and coordination and multi-stakeholder engagement. Development focussed on *quality* issues in career development sector to ensure the delivery of high-quality services. A key rationale for developing ICT use was to improve the quality and *efficiency* of career development services through more efficient use of resources and data, notably through matching technology, artificial intelligence and machine learning. Emphasis was also placed on finding new and creative ways of improving services by combining human knowledge and experience with machine-generated data and tools.

National programmes also support innovation in the delivery of career development services…

[National body] has been tasked to lead the development of a national, cross-sectoral quality framework. The process includes all stakeholders and aims to make the framework a tool for quality development and assurance and for government.

To improve the quality of private employment services, PES certifies whether they satisfy the minimum standards.

A ChatBot system that can respond to repetitive and typical questions can provide an efficient job counselling service.

In this category, *a sustained programme* of continuing professional development was provided based on identified training needs and national priorities for policy and practice. Unlike previous categories, training was now offered and can be accessed fully *online*. Here career development education is delivered as a standalone and *timetabled* subject within the curriculum, facilitated by teachers, careers professionals, teaching support staff and other external partners that include employers and further and higher education providers. Participants highlight that developing students’ career management skills helps them to better understand the job market, relate school-based learning to the workplace and develop their personal strengths to be successful in developing their careers.

Continuing professional development is based on national guidance policy and practice priorities and training needs analysis.

[Continuing profession development] training courses are not provided only in a face-to-face form, but where possible and suitable, e-training courses (including video training modules) are used.

Career information advice and guidance is delivered within the national curriculum.

### Relationship between the categories

The descriptive categories were delimited from each other and organised hierarchically according to the dimensions of variation that emerged from the data. Due to the hierarchical nature of the categories, some conceptions can be regarded as more complete and more complex than others (Åkerlind, 2005a).

Across the categories, the degree of emphasis on *innovative initiatives* shifted from desired to supported. In category 1, where innovation was conceived as initiating career development services, ambition and *desire* for innovative service initiation was expressed. In categories 2 and 3, where innovation in career development is conceived as developing demographic-based programs and professionalising the sector, innovative initiatives were *initiated* (category 2) in top-down manner and *stimulated* by empowering bottom-up processes (category 3). In the most complex category 4, innovative initiatives were *supported* by cross-sectoral collaboration and coordination and strategic stakeholder engagement.

*Development emphasis* also differed across categories. In category 1, development was mainly focussed on setting up currently non-existent *services*. In category 2, the emphasis shifted from services to developing demographic-based *programmes*. Category 3 marked a change, as the emphasis shifted from service and programme development to *professionalism*. In the most complex category 4, where the innovation in career development was conceived as exploiting cross-sectoral synergies, the development emphasis expanded on *quality* issues in the provision.

Shifts in the *rationale for developing ICT use* ranged from promoting and raising awareness of career development and related services to improving quality and efficiency. In the least complex categories, the key rationale for developing ICT use was *raising awareness* about career services (category 1) and *widening access* to guidance and information (category 2). A turning point occurred in category 3, where innovation in career development was conceived as professionalising the sector, as this marked a change to providing more *effective* client services. In the most complex category, the potential to improve the *efficiency* of career services provided the key rationale for developing ICT use.

Regarding *continuing professional development*, the transition across the categories was from acknowledged need to delivering sustained online programmes. In essence, the difference between category 1 and the other categories related to newly *acknowledged* training *needs*, whereas the more complex categories addressed the nature of training offered. A shift from acknowledged need to ad hoc training was discerned in category 2, where the innovation in career services is conceived as developing demographic-based programmes. In the most complex categories, the continuing professional development extended to *short courses* (category 3) and emphasised *sustained programmes* (category 4) based on identified training needs and national policy and practice priorities.

The *delivery of professional development* also varied across categories. In category 1, where innovation in career development was conceived as service initiation, the decisions how such training would be delivered were *not yet planned*. In category 2, professional development was delivered in *face-to-face* settings. There was a visible turning point in category 3, where innovation in career development is conceived as professionalising the sector, marking a change from face-to face delivery mode to combination of face-to-face and online, *blended* delivery. In category 4, a further important shift was observed as training was offered and delivered entirely *online*.

*Realisation of career education* ranged from pilot initiatives to timetabled subjects. In the least complex categories, selected schools or institutions were *piloting* career education (category 1) and obligatory activities were *introduced* to school curriculum (category 2). A turning point was in category 3, where innovation in career development is conceived as professionalising the sector, as this marked a change where career education became mandatory and was *integrated* in the curriculum. In the most complex category 4, a shift to a *specified time allocation* for career education as a standalone subject within the curriculum was discerned.

## Discussion

In exploring career experts’ conceptions of innovation in career development, this study identified four distinct categories of description that ranged from initiating service to exploiting cross-sectoral synergies. Six dimensions of variation were identified among the categories of description: innovative initiatives, development emphasis, rationale for developing ICT, continuing professional development, delivery of professional development and realisation of career education. While these findings generally align with earlier evidence, the present study also provides some new insights into career experts’ conceptions of innovation.

Most of the similarities to earlier studies (e.g. Amundson, 2005; Barns et al., 2020; European Commission, 2011) relate mainly to the first three categories, in which innovation in career development is conceived as initiating service, developing demographic-based programmes, and professionalising the careers sector. Like Windrum and Koch (2008) and Amundson (2005), findings in this study confirm an increased emphasis on efficacy, as well as the increasing role of ICT in career services (Kettunen & Sampson, 2019) and differences in systems of continuous professional development (OECD, 2004), including how that training is delivered (Cedefop, 2009). The findings also support earlier dominant vision that in public services innovation is a still often top-down process, which is initiated by political decisions (European Commission, 2011). In the most complex category, there is a discernible shift of emphasis towards a more collective cross-sectoral approach (e.g. Barns et al., 2020), accelerating collaborative innovation (Bommert, 2010; Torfing, 2019) to address common challenges at country level. These initiatives seek to ensure high-quality career services, with more efficient use of ICT, sustained continuing professional development programmes provided fully online and career education as a timetabled subject. Innovations are shaped through exploitation of cross-sectoral synergies. In practice, this necessitates new kinds of methods, which strengthen horizontal approaches and steering mechanisms that are adaptive to enable to respond to the rapidly changing situations.

In exploring the logical relationships between these qualitatively different conceptions of innovation, this study clarifies some key differences in career service delivery across 33 countries. The matrix presented in this article may serve as a catalyst for discussion of current and future trends. By increasing awareness and knowledge of the differences between the categories and dimensions of variation, we can stimulate dialogue that may play important role in improving innovation in career development.

The present study has some limitations. The ICCDPP symposium and this study were designed with a particular scope. The delimiting factor for the current study was the symposium’s general focus on ensuring access, integration and innovation. Participation of country teams was voluntary, and data collected at a single timepoint may not fully represent the critical factors for the entire career service sector. While the study provides a recent overview of experts’ conceptions of innovation in career service delivery, further studies are recommended in light of service changes precipitated by the onset of COVID-19. Has the COVID-19 pandemic accelerated or constrained innovation in career service delivery? The widespread transformation of in-person career services to distance services as practitioners worked remotely when offices were closed may have accelerated innovation, while the inevitable public sector budget cuts from a shrinking economy may have constrained innovation. Crisis often forces organisations to think outside the box. Dealing with pandemics, armed conflicts, and natural disasters may potentially influence the rate of innovation.
